# Value of Information: A Tool to Improve Research Prioritization and Reduce Waste

**DOI:** 10.1371/journal.pmed.1001882

**Published:** 2015-09-29

**Authors:** Cosetta Minelli, Gianluca Baio

**Affiliations:** 1 Respiratory Epidemiology, Occupational Medicine and Public Health, NHLI, Imperial College London, London, United Kingdom; 2 Department of Statistical Science, University College London, London, United Kingdom

## Abstract

In a Guest Editorial, Cosetta Minelli and Gianluca Baio explain how VOI analysis can prioritize research projects by identifying uncertainty in existing knowledge and then estimating expected benefits from reducing that uncertainty.

At a time when the scale of investments has raised justifiable concerns about the ability of ongoing research to fulfill expectations [1], the long-run sustainability of research programs will depend on demonstration of value for money. Yet, there has been remarkably little recognition of the need to formally assess research value for money in funding allocation by national governments, funding agencies, and research institutions.

Currently, research priorities are mostly decided using subjective approaches based on consensus among experts, decision makers, and other stakeholders, which tend to lack transparency and may be unduly influenced by special interest groups. More objective measures have been developed based either on the burden of disease or on variations in clinical practice [2]. Prioritization of diseases with the highest burden (morbidity, mortality, or aggregate societal costs) is useful in selecting general areas of neglect [3], but does not help identify what research should be undertaken within these areas. Prioritizing research in areas of disagreement in clinical practice can help practitioners decide between different clinical strategies and, by clarifying what is best practice, reduce variations. In the “clinical variations” method, priorities are defined based on welfare losses due to disagreement [4], with a cost-effectiveness element added in the “payback from research” method (“payback” referring to future savings as a result of the research investment) [5,6]. Setting research priorities based on variations in clinical practice, however, may not be ideal. Scientific uncertainty is not the only cause of clinical variations, which can also be due to poor implementation of research findings. Arguably, only the first should be addressed through additional research, and the second should be dealt with using more efficient means to promote good practice [7].

A decision-theoretic tool, known as “Value of Information” (VOI) [8,9], has been proposed to tackle the complexities of research prioritization in a more comprehensive way. Despite having been promoted and used for over a decade by the National Institute for Health and Care Excellence (NICE) in the United Kingdom [7], VOI is still relatively unknown to the medical scientific community.

## What Is VOI and How Does it Work?

The VOI approach consists of a set of analytic tools that can be used to assess the value of acquiring additional evidence to inform a clinical (or public health) decision [8,9]. VOI quantifies the net benefit from the improvement of population health expected from additional research against the cost of implementation. Within this framework, the value of a study is the extent to which it reduces uncertainty on a particular topic, thus potentially reducing the errors in decision-making that would have been made, had less definitive evidence been used instead.

**Fig 1:**
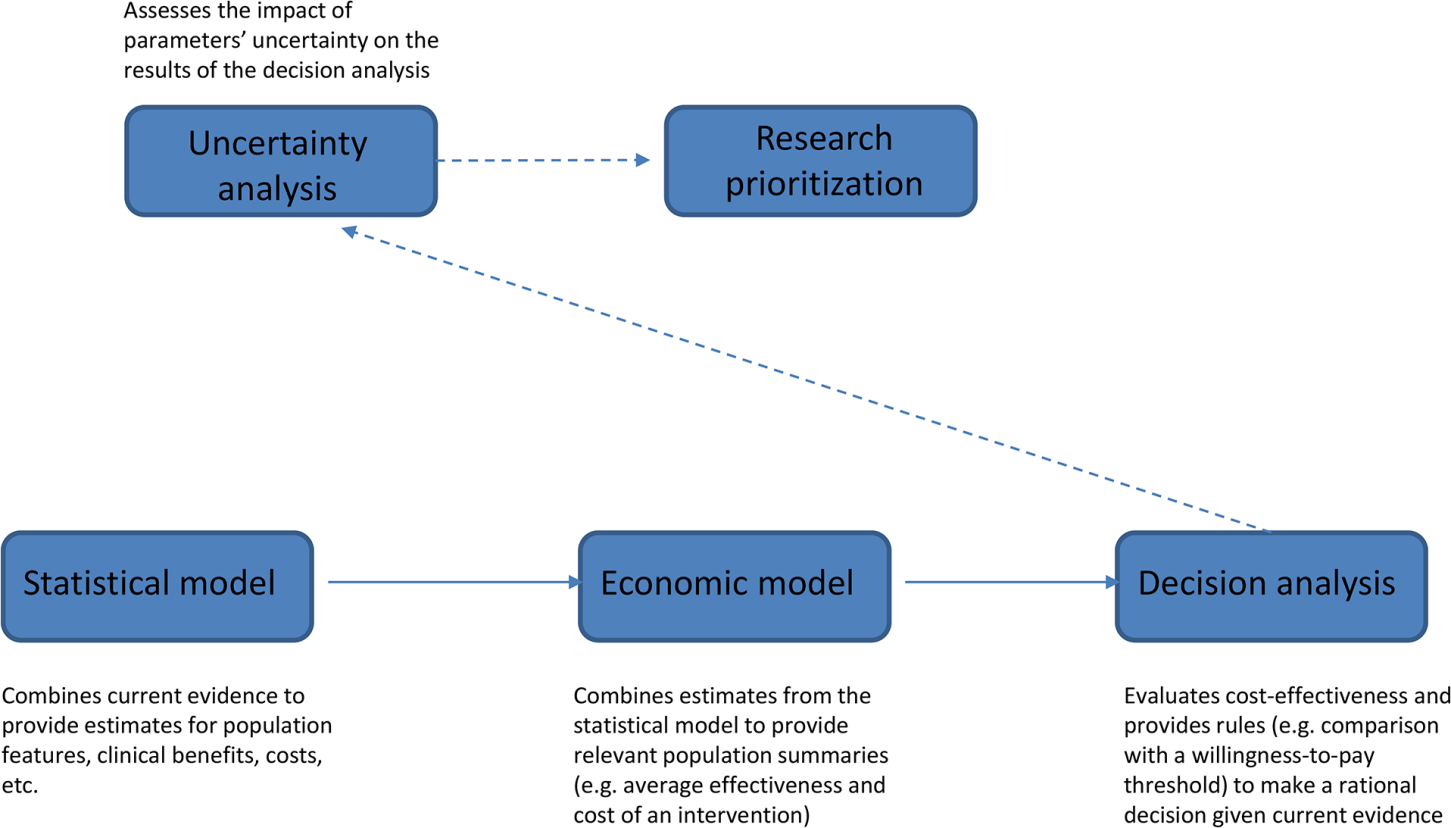
Schematic diagram illustrating the different steps of the VOI approach.

Fig 1 schematically describes the VOI approach. The starting point is a “statistical model” that estimates all relevant unknown quantities. For example, if decision-making is about implementation of a cancer screening program, relevant parameters would include sensitivity and specificity of the screening test, cancer prevalence, and health benefits (relative to mortality, morbidity, and quality of life) and costs associated with the clinical pathway with and without the proposed program. These quantities are linked by complex relationships and informed by composite sources of evidence, from published studies or resources directly available to the researcher. Evidence is synthesized using a probabilistic model, often developed within a Bayesian framework [10].

The results of the statistical model are fed into the “economic model,” which builds suitable population summaries for benefits and costs. In the example of cancer screening, we would compute the overall cost for both clinical pathways (with and without screening) by multiplying the average cost per person associated with the resources utilized (e.g., screening, diagnostic procedures, and treatments) by the expected number of users. Quality-Adjusted Life Years (QALYs), which combine changes in quantity and quality of life associated with an intervention, are often used as an average measure of clinical benefits. In our example, we would compute overall benefits of the program by multiplying QALYs gained through a successful diagnosis by the number of successful diagnoses, but we may also need to compute QALYs lost as a result of false-positive findings.

The output of the economic model provides the basis for the “decision analysis,” which applies a set of rules to determine the best course of action given current evidence. Any intervention (screening or treatment) is considered cost-effective if its cost-per-QALY does not exceed a pre-specified willingness-to-pay threshold, for example, £20,000–30,000 in the UK [11].

All parameters in the statistical model are subject to uncertainty. “Uncertainty analysis” (technically a probabilistic sensitivity analysis) assesses how much the limited knowledge about the parameters can impact the results of the decision analysis and quantifies the expected economic return of obtaining new evidence before committing to a decision. In the VOI approach, this is the crucial step informing “research prioritization.” VOI analysis may, for example, point to additional research on test sensitivity as the highest priority to reduce errors in the decision of whether to implement a screening program.

VOI analysis can be applied both to identify the crucial parameters within the appraisal of a single intervention and to compare different interventions. This kind of comparison is particularly important when allocating research funding across different topics and medical areas to maximize value for money and reduce waste.

## Challenges and Opportunities

The comprehensiveness of VOI comes at the price of a complex analytic framework based on a number of modelling choices and assumptions that may influence the results. An important choice is the perspective from which costs are to be considered. For example, costs related to productivity losses are a crucial aspect when taking a societal perspective and dealing with diseases affecting a working-age population. Productivity costs will often show large uncertainty, and a societal perspective may well point to the need to acquire this information as a priority [12]. In contrast, data on productivity costs are irrelevant from a health system perspective, in which the only costs considered are those related to health care. The results of VOI analyses also depend on the choice of health metrics. Although QALYs are far from perfect and have been criticized for relying on strong theoretical assumptions about consistency in people’s preferences [13], they represent a convenient common currency that allows comparison of health gains between different interventions and across different diseases.

VOI has classically been used for prioritization of research on health care screening and treatment interventions [14–18], but it can be equally useful in other areas, for example, risk prediction research. We are investigating its application within the Ageing Lungs in European Cohorts study (http://www.alecstudy.org), a European Commission–funded project aimed at developing a predictive risk score for chronic obstructive pulmonary disease (COPD). Although COPD is well known to be a smoking-related disease [19], there is evidence that other factors may contribute to a substantial proportion of its worldwide burden [20]. However, in addressing COPD in those who are not exposed to tobacco smoke, it is difficult to decide which other lifestyle, environmental, clinical, or genetic potential predictors should receive research priority. VOI can address this issue by evaluating the relative value for money of different research strategies according to their expected contribution in reducing the uncertainty in COPD prediction and thus minimizing prediction errors.

VOI has been used in combination with other methods at different stages of the prioritization process [21,22], but the flexible Bayesian approach [10] also allows incorporation, directly within the VOI modelling framework, of the information on which other methods are based. Such information could include disease burden and variations in clinical practice, as well as experts’ opinions on parameters for which empirical evidence is limited or not available. Similarly, patients’ preferences could be formally incorporated in VOI analyses. For example, recognition of the importance of patients’ engagement in setting research agendas has motivated initiatives such as the “priority setting partnerships” created by the James Lind Alliance, in which patients and clinicians collaborate to identify research priorities (http://www.lindalliance.org).

While VOI has been increasingly employed by regulatory agencies to inform decisions about adoption and reimbursement of treatments, its uptake by institutions prioritizing and commissioning research has been much more limited, although arguably the need to improve credibility and transparency of decisions is equally important for the latter [7]. Although VOI analyses are based on complex and computationally demanding modelling, statistical packages have been made freely available and fast methods recently developed to reduce the computational burden [23–25]. Dissemination outside the fields of health policy and health economics has also recently improved, and there are currently no real barriers to wider uptake of VOI in research prioritization.

## Abbreviations

COPD: chronic obstructive pulmonary disease
NICE: National Institute for Health and Care Excellence
QALY: Quality-Adjusted Life Year
VOI: Value of Information
